# Two Novel Measurements for the Drive-Mode Resonant Frequency of a Micromachined Vibratory Gyroscope

**DOI:** 10.3390/s131115770

**Published:** 2013-11-19

**Authors:** Ancheng Wang, Xiaoping Hu, Bing Luo, Mingming Jiang, Xiaofeng He, Kanghua Tang

**Affiliations:** College of Mechatronics and Automation, National University of Defense Technology, Changsha 410073, China; E-Mails: wangancheng@ymail.com (A.W.); ruobing@nudt.edu.cn (B.L.); toppro@yeah.net (M.J.); hexiaofeng@nudt.edu.cn (X.H.); tt_kanghua@hotmail.com (K.T.)

**Keywords:** micromachined vibratory gyroscope, drive mode, resonant frequency, one-dimensional search, golden section

## Abstract

To investigate the drive-mode resonance frequency of a micromachined vibratory gyroscope (MVG), one needs to measure it accurately and efficiently. The conventional approach to measure the resonant frequency is by performing a sweep frequency test and spectrum analysis. The method is time-consuming and inconvenient because of the requirements of many test points, a lot of data storage and off-line analyses. In this paper, we propose two novel measurement methods, the search method and track method, respectively. The former is based on the magnitude-frequency characteristics of the drive mode, utilizing a one-dimensional search technique. The latter is based on the phase-frequency characteristics, applying a feedback control loop. Their performances in precision, noise resistivity and efficiency are analyzed through detailed simulations. A test system is implemented based on a field programmable gate array (FPGA) and experiments are carried out. By comparing with the common approach, feasibility and superiorities of the proposed methods are validated. In particular, significant efficiency improvements are achieved whereby the conventional frequency method consumes nearly 5,000 s to finish a measurement, while only 5 s is needed for the track method and 1 s for the search method.

## 1. Introduction

With their advantages of low power dissipation, compact bulk, and low weight, MVGs have broad applications in military and commercial areas, such as navigation assistance, vehicle platform stabilization, consumer electronics and automation. The operation of an MVG based on the Coriolis force. First, a vibration is generated and maintained along the drive direction. If there exists an angular movement around the input axis, a Coriolis force will be formed. Then, the force causes a vibration in the sense direction. One can obtain the input angular rate by detecting the vibration along the sense axis [1–3].

Commonly, the drive mode of an MVG is excited at resonance with constant amplitude. In this case, the sense-mode vibration frequency equals the drive-mode resonant frequency [4,5]. Ideally, the drive-mode resonant frequency is a constant depending on the designed structure parameters. In practice, it is unpredictable and variable due to unavoidable fabrication inaccuracies, temperature variations or stiffness aging [6–11]. When the frequency varies, the vibration signal of the sense mode changes, and then the output of the gyroscope will drift, so the resonant frequency becomes a crucial parameter affecting the performance of the gyroscope. In order to investigate its characteristics, it is necessary to focus on the measurement of the frequency.

The conventional approach to measure the resonant frequency is by performing sweep frequency tests and spectrum analyses [12–15], which in this paper is called the sweep frequency method. Generally, to carry out a sweep frequency test, special equipment such as a frequency response analyzer [13] or dynamic signal analyzer [14], a lot of data storage, and off-line data analysis are required. The whole test process is time-consuming and inconvenient. In particular, the sweep frequency method becomes unworkable when the resonant frequency changes rapidly. The reason is that the dynamics of the system may have changed substantially during the course of measurement [11]. Therefore, more efficient methods should be explored.

In this paper, two novel resonance frequency measurement methods for the drive mode of MVGs are introduced. One, called the search method, is based on the magnitude-frequency characteristics, and utilizes a one-dimensional search technique. The other, called the track method, is based on the phase-frequency characteristics, and applies a feedback control loop. The proposed measurements can be run on-line and perform efficiently and accurately.

In the next section, we present and analyze dynamics of the MVG. Section 3 introduces basic fundamentals of the proposed methods. Section 4 discusses the performance in simulation. Section 5 presents the implemented system and experimental results. Finally, conclusions are provided in Section 6.

## 2. Dynamics of the MVG

**Figure 1.**
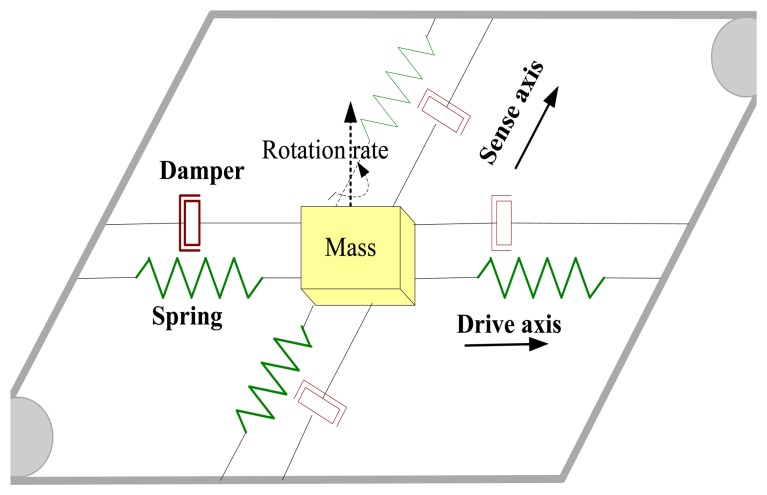
A simplified model of a MVG.

A typical MVG includes a vibration structure supported by suspensions and some electrodes. Normally, the structure oscillates freely along two orthogonal axes: the drive and sense axis. The overall system can be modeled as a mass-spring-damper structure having two degrees of freedom (2-DOF), as shown in Figure 1 [16].

In the drive direction, a controlled sinusoidal force is generated to make the mass vibrate at the drive-mode resonant frequency and achieve stable amplitude by the use of the automatic gain control (AGC) method [17]. When an angular rotation around the input axis exists, a Coriolis force will be formed. The force causes a vibration in the sense direction. Ideally, the dynamics of MVG can be described as follows [16]:

(1) $$\left[\begin{array}{cc} m & 0\\ 0 & m \end{array}\right]\;\left[\begin{array}{c} \ddot{x}\\ \ddot{y} \end{array}\right]+\left[\begin{array}{cc} D_{x} & 0\\ 0 & D_{y} \end{array}\right]\;\left[\begin{array}{c} \dot{x}\\ \dot{y} \end{array}\right]+\left[\begin{array}{cc} k_{x} & 0\\ 0 & k_{y} \end{array}\right]\;\left[\begin{array}{l} x\\ y \end{array}\right]=\left[\begin{array}{c} F_{d}\\ -2m\dot{x}\Omega \end{array}\right]$$

where *m* denotes the mass of the vibration structure, *x* and *y* are the oscillation displacements in the drive and sense axes. The parameters *k_x_*, *k_y_* represent the stiffness, and *D_x_*, *D_y_* are the damping parameters. The expression −2*mẋ*Ω represents the Coriolis force and Ω is the input angular rate around the rotation axis. *F_d_* denotes the external force that can be an electrostatic, piezoelectric or electromagnetic force.

From Equation (1), the transfer function from the force *F_d_* to the drive-mode vibration displacement *x* can be written as:

(2) $$H(s)=\frac{x}{F_{d}}=\frac{1}{ms^{2}+D_{x}s+k_{x}}=\frac{1/m}{s^{2}+(\omega_{r}/Q)s+\omega_{r}^{2}}$$

where $\omega_{r}=\sqrt{k_{x}/m}$ is the resonant frequency and $Q=\sqrt{k_{x}m}/D_{x}$ is the quality factor of the drive mode.

According to the transfer function in Equation (2), the magnitude and the phase as a function of frequency can be resolved by substituting *s* = *j*·*ω*. They are:

(3) $$A(\omega )=\left|H(j\omega )\right|=\frac{1/m}{\sqrt{{\left(\omega_{r}^{2}-\omega^{2}\right)}^{2}+\omega_{r}^{2}\omega^{2}/Q^{2}}}$$

And:

(4) $$\varphi (\omega )=∠H(j\omega )=-\arctan\frac{\omega_{r}\omega }{(\omega_{r}^{2}-\omega^{2})Q}$$

**Figure 2.**
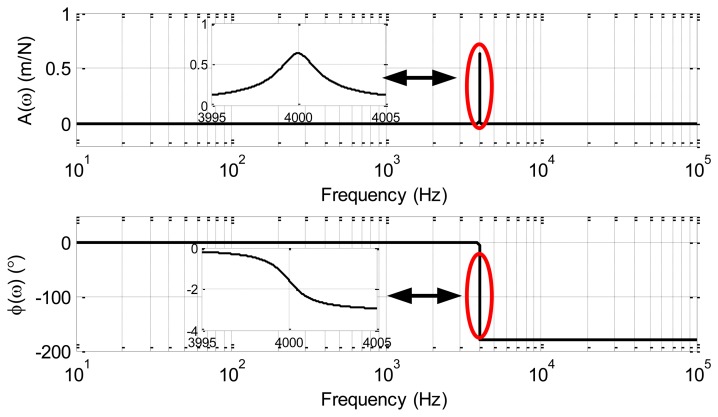
The magnitude and phase transfer function plot, with the inset showing a detailed view around the resonant frequency.

As an example, an MVG with the parameters listed in Table 1 is considered. With these values, the magnitude *A*(*ω*) and phase *φ*(*ω*) are as drawn in Figure 2. From this figure, the magnitude of the transfer function increases as the resonant frequency is approached, and reaches a maximum near the resonant frequency [5]. The phase starts at 0°, reaches −90° at the resonant frequency, and tends to −180° at high frequencies. Further analysis will be presented in Section 3.

## 3. Fundamentals of Drive-mode Resonant Frequency Measurements

Traditionally, the sweep frequency method is adopted to measure the resonant frequency of the MVG. In this method, the frequency of the input signal increases or decreases by a set step (the stepped frequency) in a given range (sweep range), and all of the response signals should be collected and recorded. In order to get a more accurate result, the stepped frequency should be small enough, and the dwell time at each frequency point should be long enough to avoid the influence of the transient. Generally, a sweep frequency test requires more than a few minutes. A problem arises that the obtained resonant frequency may not be the real frequency because the dynamics of the system may have changed substantially during the course of the measurement. In this section, two efficient methods, the search method and the track method, are introduced and their fundamentals are presented below.

### 3.1. The Search Method

Differentiating Equation (3) with respect to *ω* yields:

(5) $$A'(\omega )=-\frac{2\omega [\omega^{2}-(1-\frac{1}{2Q^{2}})\omega_{r}^{2}]}{m{\left[{\left(\omega_{r}^{2}-\omega^{2}\right)}^{2}+\omega_{r}^{2}\omega^{2}/Q^{2}\right]}^{3/2}}$$

By setting *A*′(*ω*) = 0, and considering *ω* > 0, the only frequency where the magnitude reaches its maximum value is solved to be:

(6) $$\omega_{m}=\omega_{r}\cdot \sqrt{1-1/(2Q^{2})}$$

**Figure 3.**
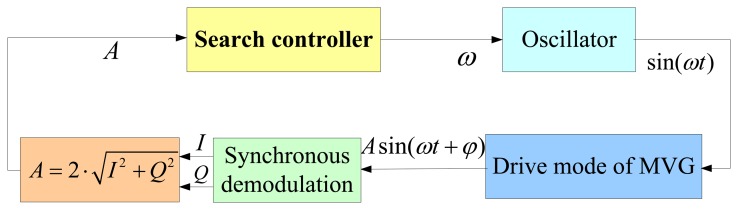
The block diagram of the proposed search method.

**Figure 4.**
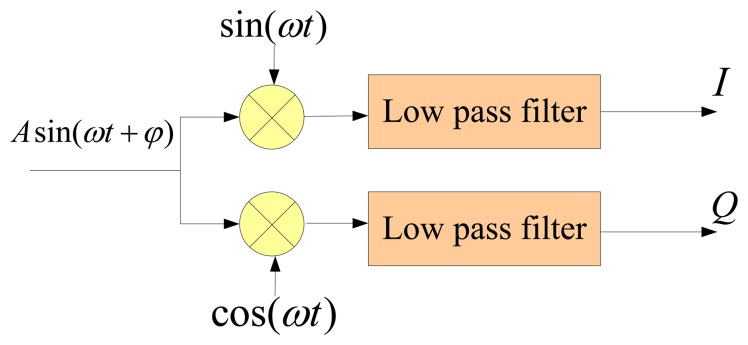
The principle diagram of the synchronous demodulation technique.

**Figure 5.**
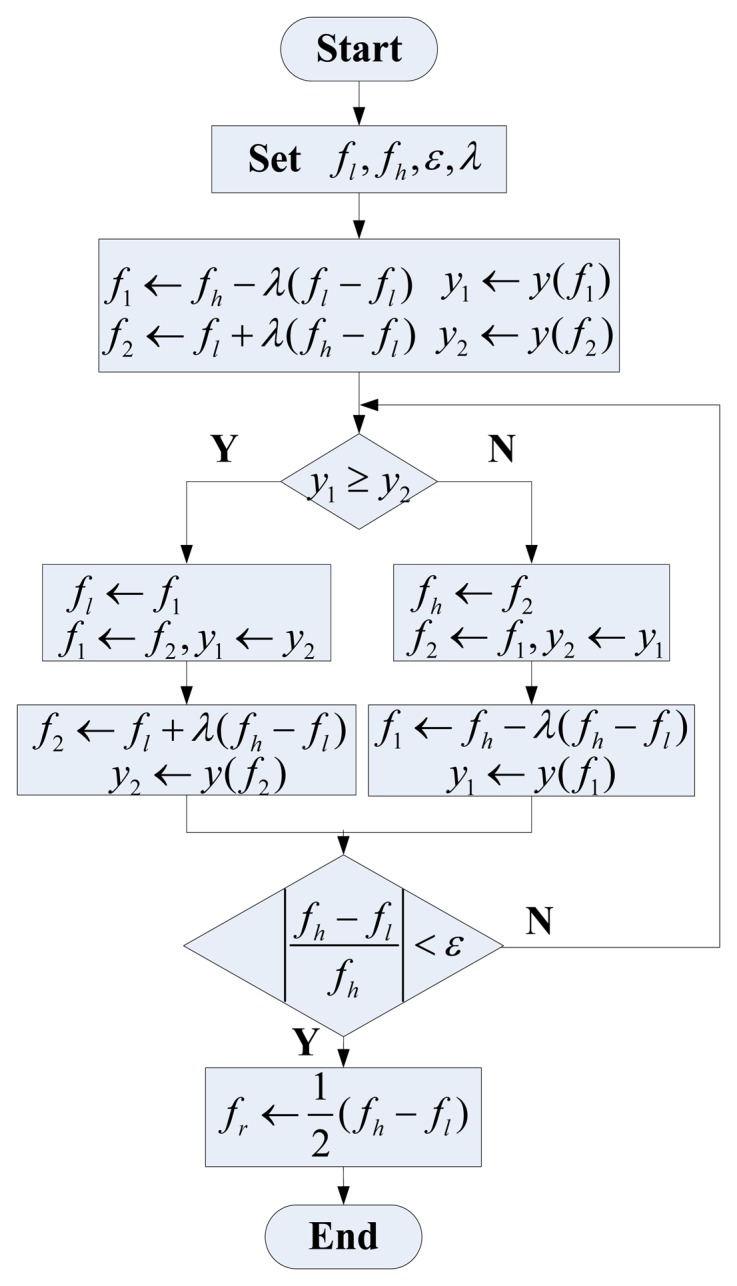
The flowchart of the applied one dimension search technique.

In practice, the MVG is normally packaged in specific vacuum level and *Q* is more than 1,000 [18]. Thus, *ω_m_* is very close to *ω_r_*. For example, if *Q* = 1,000, (*ω_r_* − *ω_m_*) ∕ *ω_r_* =2.5e-7, which implies that the magnitude-frequency function *A*(*ω*) is unimodal and approximately reaches the maximum value at the resonant frequency. Thus, to measure the resonant frequency is equivalent to finding maximum point of *A*(*ω*), and further to minimize −*A*(*ω*). According to the optimization theory, a one-dimensional search technique is a good candidate to solve the unconstrained minimization problem of a one-dimensional unimodal function [19]. Therefore, we apply it to measure the resonant frequency of the drive mode. In this paper, we name the measurement as the search method, whose principle diagram is depicted in Figure 3. The main modules and their functions are described below:

- The synchronous demodulation module is adopted to obtain the amplitude of the response signal, whose principle diagram is presented in Figure 4. Obviously:
  (7) $$\begin{array}{c} I=\text{LPF}\{\sin(\omega t+\varphi )\cdot \sin(\omega t)\}=\frac{1}{2}\cos\varphi \\ Q=\text{LPF}\{\sin(\omega t+\varphi )\cdot \cos(\omega t)\}=\frac{1}{2}\sin\varphi \end{array}$$
  Where *LPF* represents low-pass-filtering, *I*, *Q* denote the in-phase component and the quadrature component. Then the amplitude can be calculated as:
  (8) $$A=2\cdot \sqrt{I^{2}+Q^{2}}$$
- A search controller is used to control search process. It is realized based on the one-dimensional search technique, whose flowchart is shown in Figure 5. In the flowchart, *y*(*f*) = −*A*(*ω*) = −*A*(*2π*·*f*) represents function value, and *f_l_*, *f_h_* represent the start and end frequency of the search range, respectively. *ε* denotes error tolerance determining whether to end the search or not. *λ* is a constant to control the stepped frequency. Typically, when *λ* = 0.618, the algorithm is also called golden section algorithm.
- The oscillator is used to generate sine signal for a given frequency. Direct Digital Synthesizer (DDS) is a candidate to achieve the function [20].

### 3.2. The Track Method

According to Equation (4):

(9) $$\cot\varphi =\frac{(\omega^{2}-\omega_{r}^{2})Q}{\omega \omega_{r}}$$

Defining a parameter Δ*ω* to represent the difference between the current excitation signal frequency and the resonance frequency, as:

(10) $$\Delta \omega =\omega -\omega_{r}$$

Equation (9) can be rewritten as:

(11) $$\cot\varphi =\frac{(2\omega_{r}+\Delta \omega )\Delta \omega Q}{(\omega_{r}+\Delta \omega )\omega_{r}}$$

Generally, *ω_r_* is more than 10,000 and Δ*ω* is less than 200, so Δ*ω* ≪ *ω_r_*, and:

(12) $$\cot\varphi \approx \frac{2Q}{\omega_{r}}\Delta \omega$$

It is implied that cot*φ* is in direct proportion to Δ*ω*. So the value of cot*φ* can be thought as an indicator for the difference of the current excitation signal frequency and the resonance frequency, that is to say, the frequency *ω* satisfied with cot[*φ*(*ω*)] = 0 equals the resonance frequency *ω_r_*.

According to Equation (7), cot*φ* can be calculated as:

(13) cotφ=I/Q

**Figure 6.**
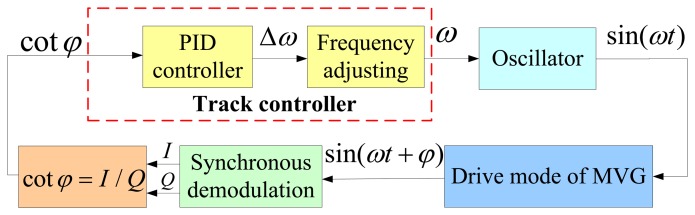
The block diagram of the track method.

Based on the above analyses, a feedback control system is proposed to measure the resonance frequency *ω_r_*, which is shown in Figure 6. The main modules and their functions are introduced as follows:

- The synchronous demodulation module here is the same as that in the search method.
- The track controller is a key module in the system, which is used to smooth the value of cot*φ* and tune the current excitation signal frequency. It should be carefully designed according to the instantaneous response and steady-state performance of the whole control system.
- The oscillator module here is the same as that in Section 3.1, which is also used to generate sine signals.

**Figure 7.**
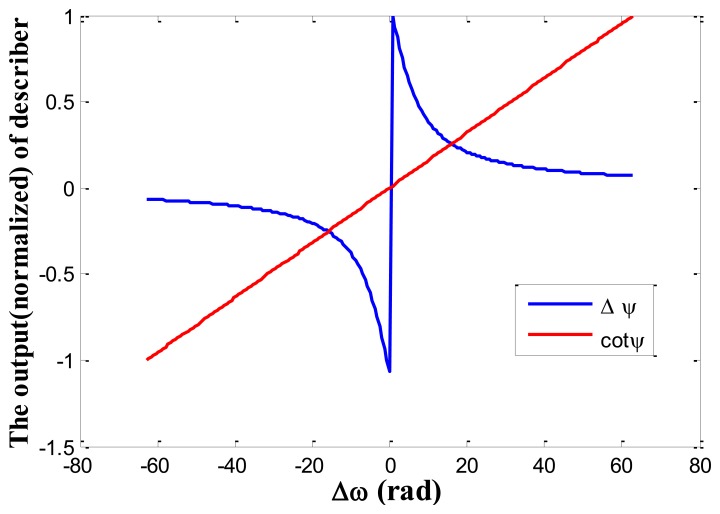
The comparison of the proposed describer (cot*φ*) and conventional PLL describer (Δ*φ*).

The closed-loop control system is similar to the phase locked loop (PLL) [21]. The main difference between the loop and conventional PLL used in drive-mode frequency keeping is the describer. In a PLL, a phase detector is required and realized according to Equation (4), so it needs an arctangent calculation process, but in the proposed control loop, the describer can be realized according to Equation (13), so the phase calculation is unneeded. Thus, as with a PLL, this loop has fewer calculations. Additionally, the describer cot*φ* has larger linearity range than Δ*φ* used in PLL, as seen in Figure 7 which shows the comparison of the proposed describer and conventional PLL describer. In the simulation, *ω_r_* = 2*π*·4,000 rad, *Q* = 2,000.

When the loop reaches stability, the frequency *ω* is locked to the resonant frequency *ω_r_*. If the resonant frequency changes, the loop will tune current frequency *ω* to track it. So the measurement is named as track method in this paper.

## 4. Simulation Analyses

In this section, simulation systems are built using Simulink to investigate the performance of the proposed methods, including the efficiency, precision, and noise resistivity. MVG parameters used here are same as those in Table 1. For the search method, we set the search range as 3,000∼5,000 Hz, error tolerance *ε* as 0.000001, and *λ* as 0.618. For the track method, we use PID controller with *P* = 32, *I* = 256 and *D* = 0, and the control period is chosen as 50 ms.

### 4.1. Simulations for the Search Method

**Figure 8.**
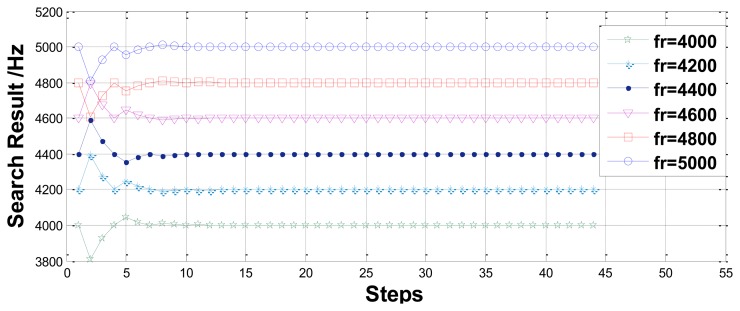
Measurement results for different resonant frequencies using the search method.

First, the precision and efficiency are studied by assigning six different resonant frequencies in the range of 4–5 kHz. The results are presented in Figure 8. The error and search steps are listed in Table 2. Clearly, the relative error of the search method is on the order of 10^−8^ and a measurement process requires only 44 search steps.

**Figure 9.**
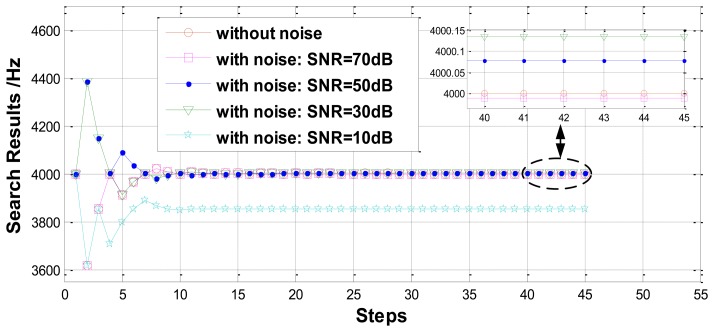
Measurement results of the search method under different level noise.

Then, the performance comparison is simulated at different noise levels. In the simulation, the resonant frequency to be measured is set as 4,000 Hz and random noise with different signal-noise-ratio (SNR) is injected into the detected amplitude of the response signal. Simulation results are plotted in Figure 9 and listed in Table 3. Obviously, the effect of the noise to the measurement precision is significant, while its effect on measurement efficiency is slight. When the SNR is 50 dB, the relative error of the search measurement is on the order of 10^−5^.

### 4.2. Simulations for the Track Method

**Figure 10.**
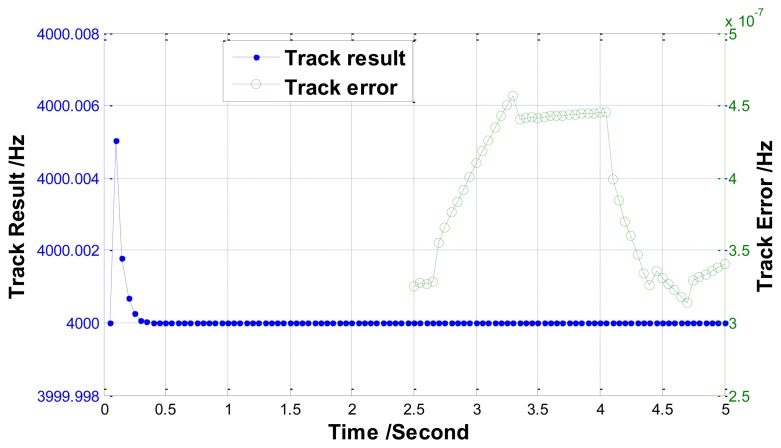
Measurement value (left) and error (right) for a constant resonant frequency (*f_r_* = 4,000 Hz) using the track method.

Three simulations are carried out to investigate the performance of the track method. In the first simulation, the resonant frequency is set as a constant (*f_r_* = 4,000 Hz). The track result and track error graphs are plotted in Figure 10. As shown in this figure, the track error approaches 3.5e-7 Hz after 5 s.

**Figure 11.**
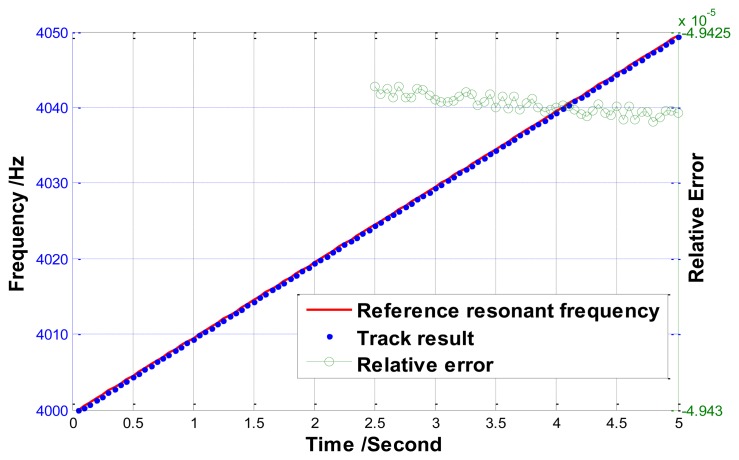
Track results when resonant frequency changes linearly from 4,000 Hz to 4,050 Hz using the track method.

The second simulation is carried out to evaluate the performance for tracking varying frequency, where the resonant frequency changes linearly with a 10 Hz/s slope from 4,000 Hz to 4,050 Hz. The results are plotted in Figure 11, which shows the relative error is about 5e-5.

**Figure 12.**
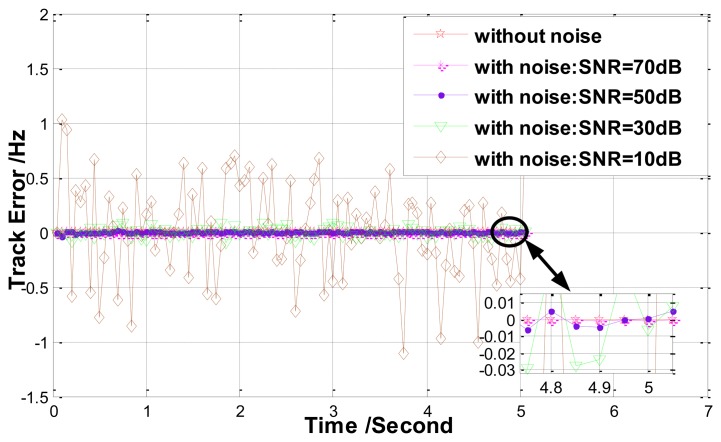
Errors of the track method under different level noise (*f_r_* = 4,000 Hz).

Finally, the performance of noise resistivity is simulated. The resonant frequency here is still set as a constant (*f_r_* = 4,000 Hz), and random noise determined by the SNR is added to the detected phase. Track errors under different level noise are presented in Figure 12. It is obvious that the track method behaves well but its measurement precision decreases. When SNR = 50 dB, the measurement error is less than 0.01 Hz.

## 5. Implementation and Experiments

**Figure 13.**
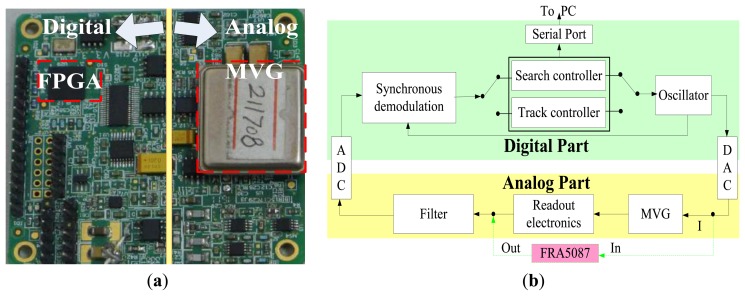
The implemented system for experiments. (**a**) The PCB. (**b**) The block diagram.

In order to study the presented methods experimentally, a measurement system is designed and implemented, as shown in Figure 13. The system includes two parts: the analog part and digital part. The analog part is mainly used for detecting the drive-mode vibration signal and filtering the noise. The digital part is implemented on an FPGA chip, including synchronous demodulation module, controller module, oscillator module and serial port module. Controllers of these two methods, search controller and track controller, are separate and programmable to switch. The measurement results are transferred to PC through a RS232 cable. As a comparison, the sweep frequency method using frequency response analysis FRA5087 is also employed, labeled in dashed lines in Figure 12b.

Two experiments are carried out to assess the actual performance of the proposed measurement methods. One is for normal temperature test where the resonant frequency varies slightly. The other is under different temperature conditions where the resonant frequency changes significantly. In each experiment, three methods are used in turn to facilitate comparison. The common parameters in the experiments are presented in Table 4.

**Figure 14.**
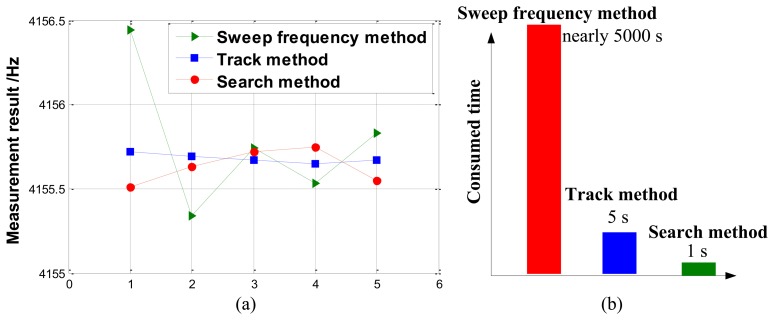
Experimental results of three methods in normal temperature. (**a**) The measurement results. (**b**) Consumed-time comparison.

In normal temperature experiment, five repeated tests are carried out. Figure 14 shows measurement results and consumed-time comparison of three methods. The statistics are presented in Table 5. Because the true value of the drive-mode resonant frequency is unknown and variable, we cannot evaluate the measurement precision directly, but the measured average values of these methods are close (the maximum difference is about 0.1 Hz), which denotes the feasibility of the proposed measurements. Besides, the track method exhibits smaller measurement variance (about 0.027 Hz), than the search method (about 0.104 Hz). As for measurement efficiency, significant differences are observed that the sweep frequency method consumes nearly 5,000 s to finish one test, but the track method only 5 s and the search method 1 s. Thus, the proposed methods yield a big efficiency improvement.

**Figure 15.**
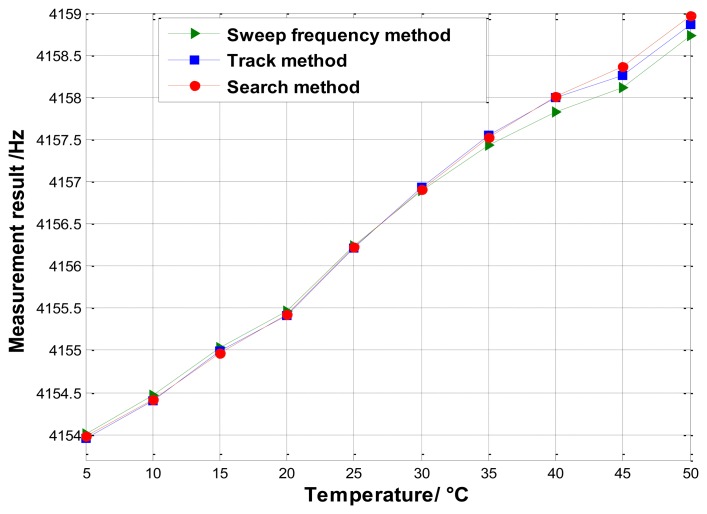
Experimental results of three methods for varying resonant frequency in different temperature.

The second experiment is carried out to measure the varying resonant frequency. The system is placed in a the temperature chamber. We make the temperature change from 5 °C to 50 °C with each temperature point kept for 1 h. The measurement results are plotted in Figure 15. Clearly, the measurements using the three different methods are approximately equivalent, which again validates the feasibility of the proposed methods.

It should be noticed that these experimental results are inadequate to evaluate the measurement precision directly. The reason is twofold. First, the true value of the drive-mode resonant frequency is unknown and variable. Second, for each experiment, three methods are conducted at different time, meaning that their true values may be different.

## 6. Conclusions

This paper focuses on the measurement for the drive-mode resonant frequency of a MVG. Two novel methods, the search method and track method, are proposed. The search method is based on the magnitude-frequency characteristics of the drive mode, and utilizes a one-dimensional search technique, while the track method is based on the phase-frequency characteristics, and applies a closed-loop control technique.

The feasibilities of the two measurement methods are validated by simulations and experiments. The simulations results show that they behave well in both measurement accuracy and noise resistivity. When the SNR of a detected signal is 50 dB, the relative error of the measurement value using the search method is only on the order of 10^−5^, and 10^−6^ for the track method.

Significant improvements in measurement efficiency are achieved by the proposed methods. Experimental results show that the traditional sweep frequency method consumes nearly 5,000 s to finish one test, while only 5 s are needed for the track method and 1 s for the search method.

Additionally, the proposed methods are easy to implement on-line because they require only a small amount of data storage. They are also applicable for resonators similar to MVGs. It should be noticed that these two novel methods fail to provide information about other parameters, for example, the Q-factor, so they are suitable for users who only want to obtain the resonant frequency of a MVG.

## Figures and Tables

**Table 1.** Example parameters for an MVG.

**Parameters**	*_m_*	*f_r_* = *ω_r_* / (2 *π*)	*Q*
Value	5 μg	4KHz	2,000

**Table 2.** Measurement errors and search steps for different resonant frequencies using the search method.

**Real Value(Hz)**	**4000**	**4200**	**4400**	**4600**	**4800**	**5000**
Measurement value(Hz)	3999.9997	4199.9997	4399.9997	4599.9997	4799.9997	4999.9997
Relative Error	−6.2479e-8	−6.2470e-8	−6.2466e-8	−6.2501e-8	−6.2530e-8	−6.2548e-8
Search steps	44	44	44	44	44	44

**Table 3.** Measurement results statistics of the search method under different level noise.

**Noise Level**	**Without Noise**	**SNR**=**70 dB**	**SNR**=**50 dB**	**SNR**=**30 dB**	**SNR**=**10 dB**
Measurement value (Hz)	3999.9997	3999.9886	4000.0786	4000.1357	3852.6087
Relative error	−6.248e-8	−2.8500e-6	1.9650e-5	3.3925e-5	−0.0368
Search steps	45	45	45	45	45

**Table 4.** Parameters in the experiments.

**Parameters**	**Search Method**				**Track Method**				**Sweep Frequency Method**		

	**Range**	*ε*	*λ*	**Stepped time**	**P**	**I**	**D**	**Control period**	**Range**	**Stepped frequency**	**dwell time**
Value	3.5–4.5 KHz	0.001	0.618	50 ms	32	256	0	50 ms	3.5–4.5 KHz	0.01 Hz	50 ms

**Table 5.** Statistics of experimental results in normal temperature.

	**1**	**2**	**3**	**4**	**5**	**Average**	**Standard variance**
Sweep frequency Method(Hz)	4156.44	4155.34	4155.74	4155.53	4155.83	4155.78	0.4170
Track method(Hz)	4155.72	4155.69	4155.67	4155.65	4155.67	4155.68	0.0265
Search method(Hz)	4155.51	4155.63	4155.72	4155.75	4155.55	4155.63	0.1040
